# Serum Insulin, Insulin-Like Growth Factor-1, Testosterone and Lipid Profile Levels in Benign Prostatic Hyperplasia and Prostate Cancer at Diagnosis

**DOI:** 10.7759/cureus.75342

**Published:** 2024-12-08

**Authors:** Afreen Khan, Esha Sarkar, Anu Chandra, Syed Tasleem Raza, Abbas A Mahdi, S N Sankhwar, Preeti Agarwal, Avneet Gupta

**Affiliations:** 1 Biochemistry, Era's Lucknow Medical College and Hospital, Era University, Lucknow, IND; 2 Urology, King George's Medical University, Lucknow, IND; 3 Pathology, King George's Medical University, Lucknow, IND; 4 Urology, Precision Urology Hospital, Lucknow, IND

**Keywords:** benign prostatic hyperplasia, gleason score, insulin, insulin-like growth factor, lipid profile, prostate cancer

## Abstract

Background and objectives: Prostate cancer is the second most frequently diagnosed cancer in men aged 65 years and older globally. The association of prostate cancer with deranged lipid profile and insulin levels is inconsistent and not well understood. This study aimed to analyze the serum levels of lipids, insulin, insulin-like growth factor-1 (IGF-1) and testosterone and to identify their association with the risk of benign prostatic hyperplasia, prostate cancer and its grading.

Materials and methods: This case-control study includes 150 individuals. Cases were 50 newly diagnosed benign prostatic hyperplasia (BPH) and 50 histologically confirmed prostate adenocarcinoma patients. Fifty age-matched disease-free controls were included. Results were analyzed using descriptive statistics and summarized as mean ± standard deviation. ANOVA was used to determine statistically significant differences between two or more categorical groups. Chi-square was used to determine the association between variables of interest.

Results: Data showed that serum insulin and IGF-1 were significantly elevated in prostate cancer and BPH, the highest being in the prostate cancer group, and had a significant positive association with prostate cancer Gleason score and grade. However, lipid profile had non-significant association with prostate cancer Gleason score and grade.

Conclusion: This study confirms the association of insulin and IGF-1 with BPH and prostate cancer Gleason score and grade.

## Introduction

Prostate cancer is the second most frequently diagnosed cancer in men aged 65 years and older and the third leading cause of cancer-related mortality globally. In India, approximately 43,691 new cases with an incident rate of one in 125 were reported in 2022 with an expected rise of over 47,000 cases by 2025 as reported by the Indian Council of Medical Research (ICMR) National Cancer Registry Programme 2022 [1]. Prostate cancer growth and development rely on androgens (testosterone and dihydrotestosterone) which leads to differentiation and proliferation of epithelial prostate cells via androgen receptor signaling, thus making way for androgen deprivation therapy as one of the most popular therapeutic approaches in prostate cancer treatment. However, mutations in androgen receptor gene proceed to androgen-independent prostate cancer growth. Concurrently, other molecular pathways are also dysregulated which include growth factors such as insulin-like growth factor-1 and insulin, which contribute to tumorigenesis [2]. Recent epidemiological studies tried to establish association of insulin-like growth factor-1 and insulin with prostate cancer incident and mortality; however, the reported literature is inconsistent. Insulin and insulin-like growth factor-1 bind non-selectively to their receptors and act as mitogens. Furthermore, chronic hyperinsulinemia may stimulate carcinogenesis via binding with both insulin receptor and insulin-like growth factor-1 receptor. Other factors such as diet, physical activity or body mass index could also contribute to prostate carcinogenesis [3,4]. The aim of our present study was to estimate serum insulin, insulin-like growth factor-1, testosterone and lipid levels in benign prostatic hyperplasia, prostate cancer and control group and establish their association with risk of benign prostatic hyperplasia and prostate cancer and its severity at the time of diagnosis.

## Materials and methods

Study population

This case-control study was conducted on a total of 150 individuals recruited from June 2020 to June 2023. Patients having prostate-specific antigen levels greater than 4 ng/mL underwent a 12-core transrectal ultrasound-guided (TRUS) biopsy before transurethral resection of prostate (TURP) to rule out prostate cancer. Clinical data was obtained from 50 benign prostatic hyperplasia and 50 prostate cancer patients who underwent transurethral resection of prostate at the Department of Urology, King George's Medical University and Precision Urology Hospital. Personal history (smoking, alcohol consumption and exercise status) and medical history (diabetes, hypertension, etc.) were taken based on standardized questionnaire and interview. Body mass index was calculated using formula: weight (kg)/height^2^ (m^2^). Patients having history of diabetes mellitus, coronary artery disease, hepatic/renal diseases or on medications for testosterone replacement and androgen deprivation therapy were excluded. Fifty age‑matched disease‑free males, without any complications, were recruited as controls.

Specimen collection and laboratory assays

Two milliliter of blood was withdrawn from the antecubital vein and collected in plain vial following overnight fasting. Serum was separated by centrifugation at 3,000 rpm for 15 minutes at room temperature. Serum samples were stored at -80°C until use. Serum lipids (total cholesterol, high-density lipoprotein cholesterol and triglycerides) were measured enzymatically on semi-autoanalyzer. Friedwald’s formula was used to calculate serum very low‑density lipoprotein cholesterol (VLDL‑C) and low‑density lipoprotein cholesterol (LDL‑C). Insulin, insulin-like growth factor-1 and testosterone were measured using DRG ELISA kits (Catalogue No. EIA-2935, EIA-4140 and EIA-1559, respectively) according to the manufacturer’s protocol.

Statistical analysis

This was a case-control study. The results were analyzed using descriptive statistics, making comparisons among various groups. Qualitative variables were summarized as number and relative frequency (%), whereas quantitative variables were summarized as mean ± standard error, respectively. Chi-square test was used to test the association between variables. Analysis of variance (ANOVA) was used to determine the statistically significant difference among benign prostatic hyperplasia, prostate cancer and control groups. The Tukey post hoc test was used to identify pairwise differences between means in a one-way ANOVA. Multinomial logistic regression analysis was done to assess the relationship of study parameters with the risk of benign prostatic hyperplasia and prostate cancer. Variables that showed p-value <0.05 were selected for multivariate analysis. In addition, the association of collected variables with benign prostatic hyperplasia and prostate cancer risk was analyzed. Finally, the levels of insulin, insulin-like growth factor-1, prostate-specific antigen and testosterone were compared with physical activity and addiction habits. All statistical analysis were performed using Statistical Package for Social Sciences (SPSS) version 26 (IBM Corp., Armonk, N.Y., USA) and GraphPad Prism 9 (GraphPad Software, Boston, USA). The significance level was taken as p<0.05.

Ethics statement

Study was approved by the Institutional Ethics Committee, King George's Medical University (ECR/262/Inst/UP/2013/RR-19) and Era University (ELMC&H/R_Cell/EC/2020/121A). A written informed consent was obtained from each subject.

## Results

Baseline characteristics and biochemical parameters

**Figure 1:**
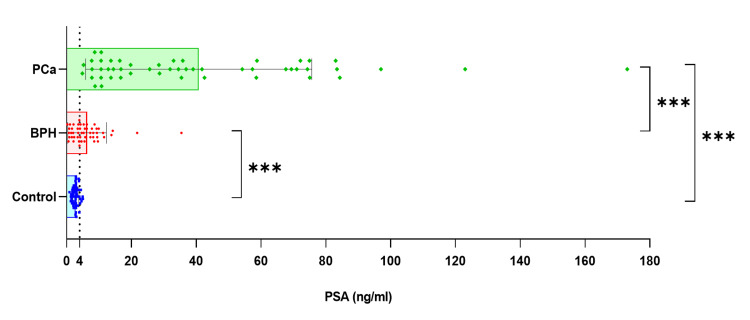
Graph showing serum PSA levels in BPH, prostate cancer and controls. *** represents highly significant results (p value<0.001). Box and error bars represent mean±SD of each group, whereas dots represents individual study subject. PCa: prostate cancer, PSA: prostate-specific antigen, BPH: benign prostatic hyperplasia.

Mean age and body mass index among prostate cancer, benign prostatic hyperplasia and control group showed significant statistical difference. The mean serum prostate-specific antigen levels were 2.78 ± 0.96 ng/mL, 6.30 ± 5.94 ng/mL and 40.75 ± 34.90 ng/mL in controls, benign prostatic hyperplasia and prostate cancer, respectively (Figure 1). Serum insulin and insulin-like growth factor-1 were significantly elevated in benign prostatic hyperplasia and prostate cancer group compared to controls with highest values in prostate cancer group (p-value <0.002 for insulin, <0.001 for insulin-like growth factor-1 and <0.014 for testosterone). Serum lipids (total cholesterol, triglycerides, low-density lipoprotein and very low-density lipoprotein) were also elevated in prostate cancer and benign prostatic hyperplasia patients compared to controls. However, the difference was non-significant. High-density lipoprotein and testosterone were significantly deceased in the prostate cancer and benign prostatic hyperplasia group compared to controls (p-value <0.035). Additional baseline characteristics and biochemical parameters of the cases and controls included in this study are presented in Tables 1-3.

**Table 1:** Baseline characteristics of the enrolled benign prostatic hyperplasia and prostate cancer patients and control groups. BPH: benign prostatic hyperplasia, BMI: body mass index.

Parameters		Control	Cases	
		(n=50)	BPH (n=50)	(n=50)
Age (years), n (%)	<55	14 (28%)	6 (12%)	2 (4%)
	55-64	21 (42%)	19 (38%)	10 (20%)
	65-74	15 (30%)	19 (38%)	22 (44%)
	≥75	0	6 (12%)	16 (16%)
BMI (kg/m^2^), n (%)	<25	44 (88%)	37 (74%)	40 (80%)
	≥25	6 (12%)	13 (26%)	10 (20%)
Smoking status (n)	Yes (%)	17 (34%)	21 (42%)	19 (38%)
	No (%)	33 (66%)	29 (58%)	31 (62%)
Alcohol consumption	Yes (%)	15 (30%)	18 (36%)	20 (40%)
	No (%)	35 (70%)	32 (64%)	30 (60%)
Physical activity	Not very active	6 (12%)	9 (18%)	12 (24%)
	Moderately active	29 (58%)	33 (66%)	32 (50%)
	Very active	15 (30%)	8 (16%)	6 (12%)
Diet pattern	Vegetarian	36 (72%)	39 (78%)	31 (63%)
	Non-vegetarian	14 (28%)	11 (22%)	19 (38%)
Hypertension	Yes (%)	11 (22%)	23 (46%)	26 (52%)
	No (%)	39 (78%)	27 (42%)	24 (48%)

**Table 2:** Intergroup comparison of study variables among control, benign prostatic hyperplasia and prostate cancer cases. Bold values represent significant results (p<0.05). BMI: body mass index, T.CHO: total cholesterol, TG: triglyceride, HDL: high-density lipoprotein, LDL: low-density lipoprotein, VLDL: very low-density lipoprotein, PSA: prostate-specific antigen, IGF-1: insulin-like growth factor-1, BPH: benign prostatic hyperplasia, PCA: prostate cancer.

Variable	Control		BPH		PCA		ANOVA	
	Mean	SD	Mean	SD	Mean	SD	F-value	p-value
Age	55.02	9.17	64.74	9.58	70.24	8.44	36.05	<0.001
BMI	20.96	3.13	22.53	3.77	22.25	3.04	3.14	0.046
T. CHO	171.75	38.11	184.29	50.57	188.19	39.30	1.99	0.140
TG	152.20	65.25	157.42	54.57	169.86	60.27	1.14	0.324
HDL	53.33	11.41	50.40	6.49	48.60	8.79	3.43	0.035
LDL	87.98	36.61	102.40	47.76	105.63	39.86	2.54	0.082
VLDL	30.44	13.05	31.48	10.91	33.97	12.05	1.14	0.324
PSA	2.78	0.96	6.30	5.94	40.75	34.90	52.65	<0.001
Insulin	13.29	4.42	14.81	4.37	17.54	8.44	6.33	0.002
IGF-1	335.30	35.22	341.71	22.64	360.47	27.03	10.33	<0.001
Testosterone	3.99	1.22	3.57	1.92	3.11	1.22	4.40	0.014

**Table 3:** Post hoc paired comparisons of study variables between control, benign prostatic hyperplasia and prostate cancer groups. Bold values represent significant results (p<0.05). BMI: body mass index, HDL: high-density lipoprotein, PSA: prostate-specific antigen, IGF-1: insulin-like growth factor-1, BPH: benign prostatic hyperplasia, PCA: prostate cancer.

Dependent Variable	BPH vs PCA		BPH vs Control		PCA vs Control	
	Mean Diff.	p-value	Mean Diff.	p-value	Mean Diff.	p-value
Age	-5.50	0.008	9.72	<0.001	15.22	<0.001
BMI	0.27	0.912	1.56	0.053	1.29	0.132
HDL	1.80	0.585	-2.93	0.246	-4.73	0.028
PSA	-34.40	<0.001	3.52	0.666	37.97	<0.001
Insulin	-2.72	0.066	1.53	0.420	4.25	0.002
IGF-1	-18.70	0.004	6.41	0.507	25.17	<0.001
Testosterone	0.46	0.267	-0.41	0.340	-0.88	0.010

Relationship of biochemical parameter with benign prostatic hyperplasia and prostate cancer

**Figure 2:**
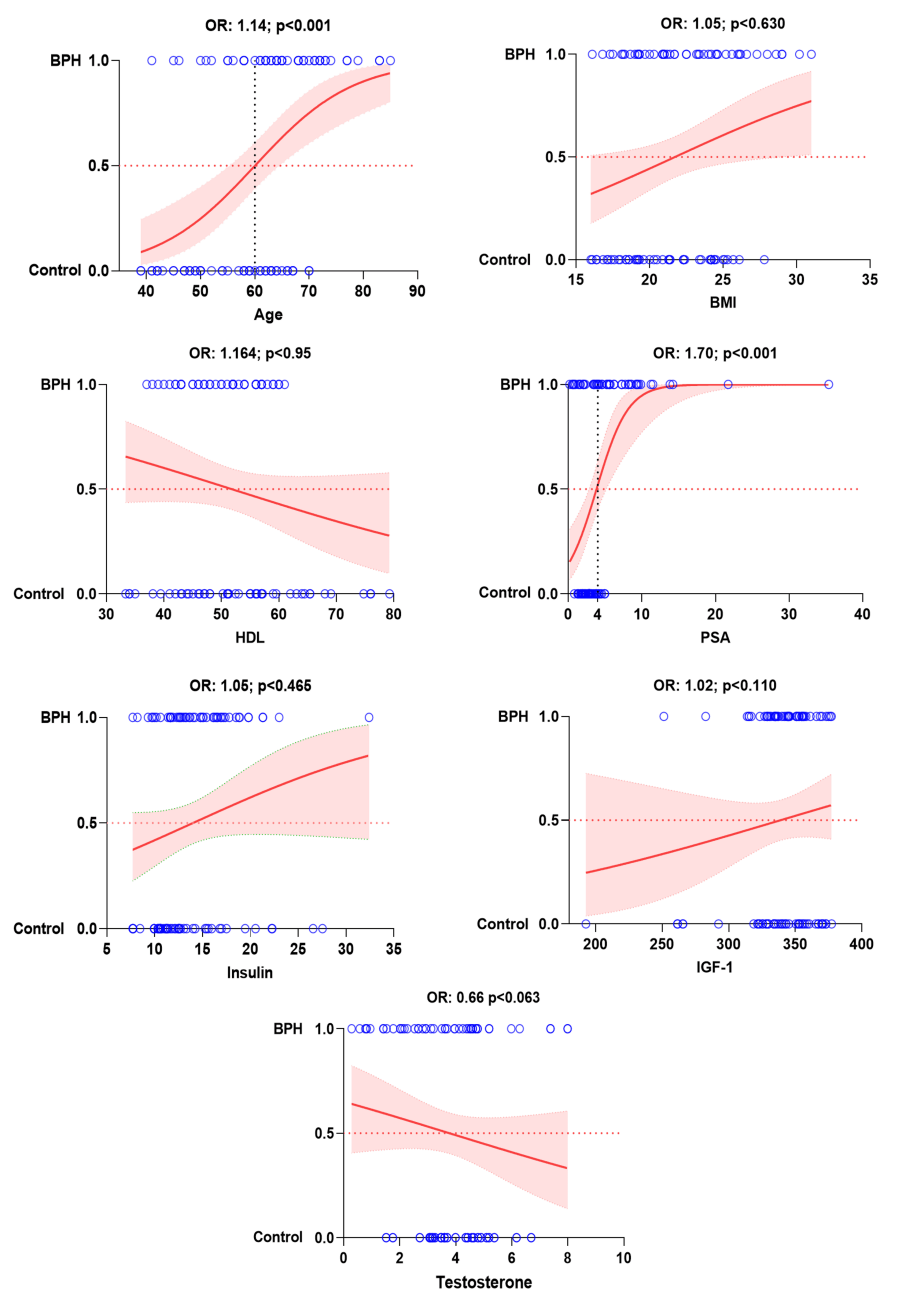
Multinomial regression analysis logistic plot showing relationship of BPH with significant influencing parameters. BPH: benign prostatic hyperplasia, BMI: body mass index, HDL: high-density lipoprotein, PSA: prostate-specific antigen, IGF-1: insulin-like growth factor-1.

**Figure 3:**
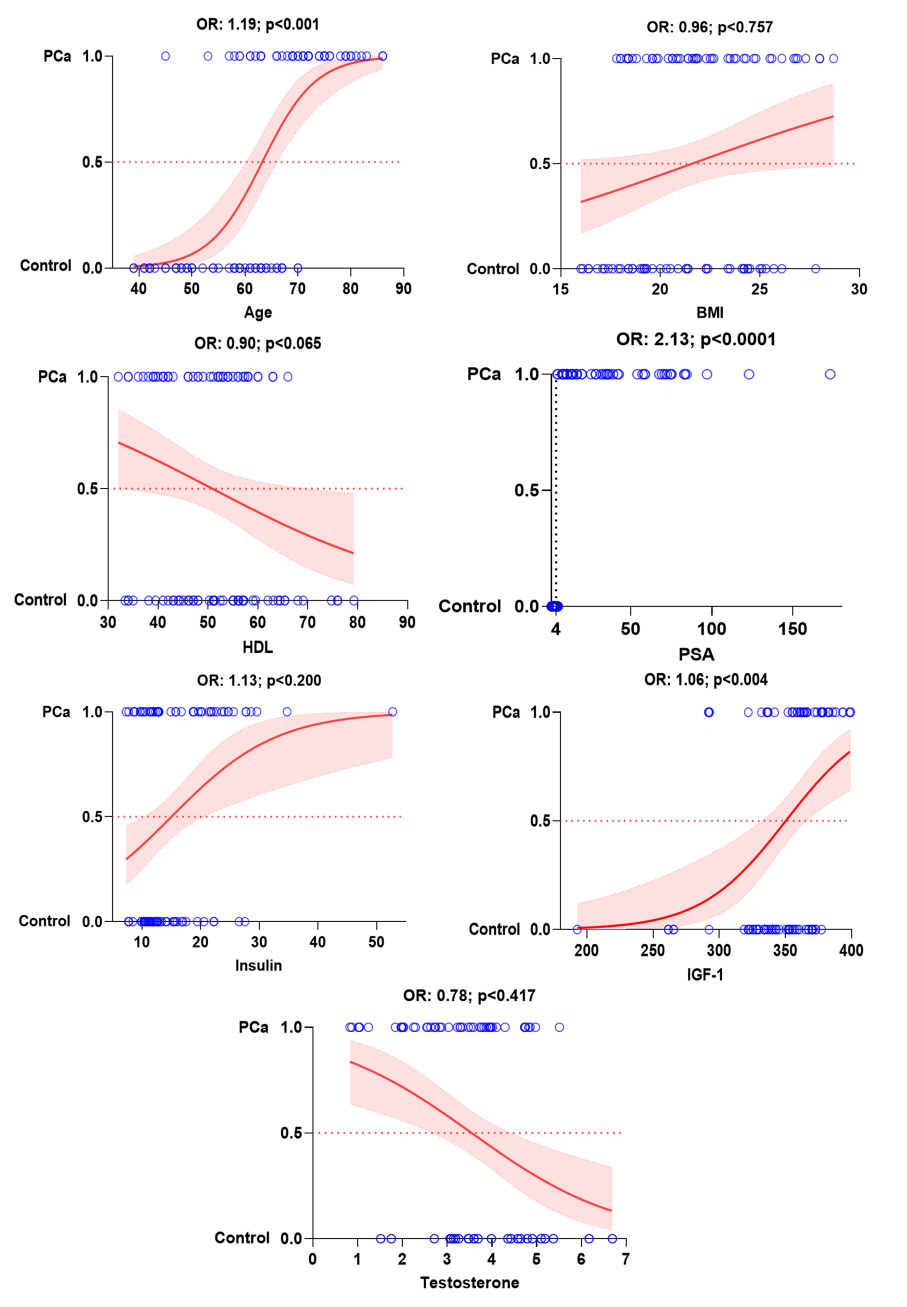
Multinomial regression analysis logistic plot showing relationship of prostate cancer with significant influencing parameters. PCa: prostate cancer, BMI: body mass index, HDL: high-density lipoprotein, PSA: prostate-specific antigen, IGF-1: insulin-like growth factor-1.

Multinomial regression analysis of benign prostatic hyperplasia and prostate cancer with biochemical parameters showed that each additional year of age leads to an expected increase 14% in the chances of benign prostatic hyperplasia (OR: 1.14; 95% CI; p<0.001) and 19% increase in the chances of prostate cancer (OR: 1.19; 95% CI; p<0.001). Similarly, with each unit increase in serum prostate-specific antigen levels, chance of occurrence of benign prostatic hyperplasia (OR: 1.70; 95% CI; p<0.001) and prostate cancer (OR: 2.13; 95% CI; p<0.001) increases by 70% and 113%, respectively considering all other parameters to be constant. Each unit increase in serum insulin-like growth factor-1 levels increases the chances of prostate cancer by 6% (OR: 1.06; 95% CI; p<0.004). Multiple logistic regression of all other parameters with benign prostatic hyperplasia and prostate cancer is non-significant (Figures 2, 3 and Table 4).

**Table 4:** Multinomial regression analysis showing relationship of BPH and prostate cancer with significant influencing parameters. Bold values represent significant results (p<0.05). BMI: body mass index, HDL: high-density lipoprotein, PSA: prostate-specific antigen, IGF-1: insulin-like growth factor-1, PCA: prostate cancer, BPH: benign prostatic hyperplasia.

Dependent : BPH wrt Control	B	SE	p-value	Exp(B)
Intercept	-13.73	5.29	0.009	
Age	0.13	0.04	<0.001	1.14
BMI	0.05	0.10	0.630	1.05
HDL	-0.05	0.03	0.164	0.95
PSA	0.53	0.15	<0.001	1.70
Insulin	0.05	0.07	0.465	1.05
IGF-1	0.02	0.01	0.110	1.02
Testosterone	-0.41	0.22	0.063	0.66
Dependent: PCA wrt control	B	SE	p-value	Exp(B)
Intercept	-29.50	8.78	0.001	
Age	0.17	0.05	0.001	1.19
BMI	-0.04	0.14	0.757	0.96
HDL	-0.11	0.06	0.065	0.90
PSA	0.76	0.16	<0.001	2.13
Insulin	0.12	0.10	0.200	1.13
IGF-1	0.06	0.02	0.004	1.06
Testosterone	-0.25	0.30	0.417	0.78

Biochemical parameters with prostate cancer aggressiveness: Gleason score and grade

Serum prostate-specific antigen levels show significant positive correlation with age (p-value <0.001). However, the correlation was non-significantly positive with body mass index and lipid profile parameters. Similarly, non-significant correlation of insulin-like growth factor-1 and insulin was observed with body mass index and lipid profile parameters (Table 5).

**Table 5:** Correlation of PSA, insulin, IGF-1 and testosterone with other influencing parameters in prostate cancer group. Bold values represent significant results (p<0.05). BMI: body mass index, T.CHO: total cholesterol, TG: triglyceride, HDL: high-density lipoprotein, LDL: low-density lipoprotein, VLDL: very low-density lipoprotein, PSA: prostate-specific antigen, IGF-1: insulin-like growth factor-1.

Pearson Correlation	PSA		Insulin		IGF-1		Testosterone	
	r-value	p-value	r-value	p-value	r-value	p-value	r-value	p-value
Age	0.295	<0.001	0.212	0.009	0.117	0.152	-0.027	0.744
BMI	-0.030	0.717	0.010	0.905	0.067	0.413	-0.258	0.001
T. CHO	0.077	0.349	-0.017	0.834	0.001	0.995	-0.140	0.088
TG	0.048	0.562	0.036	0.664	0.046	0.574	-0.144	0.079
HDL	-0.135	0.100	-0.066	0.423	-0.110	0.181	0.191	0.019
LDL	0.095	0.246	-0.014	0.870	0.011	0.889	-0.145	0.077
VLDL	0.048	0.562	0.036	0.664	0.046	0.574	-0.144	0.079

Serum total cholesterol, triglyceride, low-density lipoprotein, very low-density lipoprotein and prostate Specific antigen levels showed significant positive correlation, and serum high-density lipoprotein showed significant negative correlation with prostate cancer Gleason score and grade. Serum insulin and insulin-like growth factor-1 showed non-significant positive correlation, whereas testosterone showed non-significant negative correlation with prostate cancer Gleason score and grade (Table 6).

**Table 6:** Correlation of prostate cancer Gleason score and grade with influencing parameters. Bold values represent significant results (p<0.05). BMI: body mass index, T.CHO: total cholesterol, TG: triglyceride, HDL: high-density lipoprotein, LDL: low-density lipoprotein, VLDL: very low-density lipoprotein, PSA: prostate-specific antigen, IGF-1: insulin-like growth factor-1.

Among Prostate Cancer Cases				
Spearman Correlation	Gleason Score		Cancer Grade	
	r-value	p-value	r-value	p-value
Age	-0.265	0.006	-0.268	0.059
BMI	0.290	0.040	0.280	0.048
T. CHO	0.408	0.003	0.413	0.003
TG	0.336	0.017	0.281	0.048
HDL	-0.350	0.012	-0.327	0.021
LDL	0.394	0.004	0.394	0.005
VLDL	0.336	0.016	0.281	0.048
PSA	0.256	0.071	0.304	0.031
Insulin	0.178	0.215	0.157	0.278
IGF-1	0.110	0.443	0.078	0.589
Testosterone	-0.070	0.629	-0.035	0.810

Activities and addictions

Cases having family history of prostate cancer have no significant difference in serum prostate-specific antigen, testosterone, insulin and insulin-like growth factor-1 levels compared to cases with no family history for prostate cancer. Similar findings were observed with smoking status, alcohol consumption, diet and history of hypertension. However, a significant difference of serum insulin levels was observed with physical activity, serum insulin levels being significantly elevated in cases who are not very active and lower levels in cases who are moderately or very active (Tables 7-10).

**Table 7:** Comparison of PSA levels with activities and addictions. PSA: prostate-specific antigen, HTN: hypertension. p<0.05 is considered as significant.

Variable		PSA		Significance	
		Mean	SD	t or F value	p value
Family history	No	23.73	31.93	t=0.15	p=0.081
	Yes	22.51	21.63		
Smoking	No	15.58	26.10	t=0.61	p=0.542
	Yes	18.33	27.59		
Alcohol	No	15.14	25.40	t=0.91	p=0.363
	Yes	19.29	28.73		
Physical activity	Not very active	21.09	36.20	F=1.13	p=0.327
	Moderately active	17.15	24.84		
	Very active	10.67	21.05		
Diet	Vegetarian	14.94	24.87	t=1.20	p=0.236
	Non-vegetarian	20.62	30.32		
HTN	No	15.96	27.71	t=0.36	p=0.721
	Yes	17.55	25.10		

**Table 8:** Comparison of insulin level with activities and addictions. PSA: prostate-specific antigen, HTN: hypertension. p<0.05 is considered as significant.

Variable		Insulin		Significance	
		Mean	SD	t or F value	p value
Family history	No	16.54	7.08	t=1.17	p=0.244
	Yes	14.41	5.23		
Smoking	No	15.31	6.78	t=0.25	p=0.807
	Yes	15.05	5.36		
Alcohol	No	15.09	6.64	t=0.32	p=0.747
	Yes	15.44	5.56		
Physical activity	Not very active	18.31	9.10	F=4.80	p=0.010
	Moderately active	14.21	5.21		
	Very active	15.59	5.41		
Diet	Vegetarian	14.65	6.11	t=1.73	p=0.085
	Non-vegetarian	16.58	6.48		
HTN	No	15.34	6.80	t=0.31	p=0.758
	Yes	15.02	5.45		

**Table 9:** Comparison of IGF-1 level with activities and addictions. IGF-1: insulin-like growth factor-1, HTN: hypertension.

Variable		IGF-1		Significance	
		Mean	SD	t or F value	p value
Family history	No	351.39	27.49	t=0.25	p=0.806
	Yes	349.64	21.99		
Smoking	No	343.30	33.17	t=1.32	p=0.189
	Yes	350.07	25.20		
Alcohol	No	344.54	33.06	t=0.70	p=0.448
	Yes	348.18	25.37		
Physical activity	Not very active	341.89	46.85	F=0.28	p=0.753
	Moderately active	346.93	26.53		
	Very active	345.91	23.72		
Diet	Vegetarian	344.74	28.06	t=0.67	p=0.501
	Non-vegetarian	348.44	36.00		
HTN	No	342.78	33.25	t=1.48	p=0.140
	Yes	350.28	25.66		

**Table 10:** Comparison of testosterone level with activities and addictions. HTN: hypertension.

Variable		Testosterone		Significance	
		Mean	SD	t or F value	p value
Family history	No	3.32	1.72	t=0.33	p=0.741
	Yes	3.43	1.07		
Smoking	No	3.67	1.49	t=1.25	p=0.215
	Yes	3.35	1.58		
Alcohol	No	3.57	1.46	t=0.21	p=0.833
	Yes	3.52	1.65		
Physical activity	Not very active	3.32	2.09	F=0.99	p=0.375
	Moderately active	3.69	1.34		
	Very active	3.33	1.49		
Diet	Vegetarian	3.58	1.40	t=0.37	p=0.715
	Non-vegetarian	3.48	1.81		
HTN	No	3.58	1.49	t=0.28	p=0.784
	Yes	3.51	1.58		

## Discussion

Age is considered to be a risk factor for both benign prostatic hyperplasia and prostate cancer, with an average of 66 years at the time of diagnosis. Evidence that the risk of benign prostatic hyperplasia and prostate cancer increases with increasing age had already been established in some community-based studies as prostate blood supply damages and levels of steroid-sex hormones change with age. Higher clinical stage, biopsy grade and PSA velocity in patients older than 70 years were reported in the randomized clinical trial ISRCTN54449243 [5]. Our results are in line with these studies.

Insulin resistance or hyperinsulinemia might be an important etiological factor in the pathogenesis of benign prostatic hyperplasia and prostate cancer. In this study, we observed significantly elevated insulin and insulin-like growth factor-1 levels in both benign prostatic hyperplasia and prostate cancer groups. The elevation was noticeable in the prostate cancer group. Our research also observed positive association of insulin and insulin-like growth factor-1 levels with Gleason score and grade of prostate cancer. Previous studies also reported similar findings [6-8]. Saboori et al. performed a meta-analysis to analyze serum insulin levels and insulin resistance in prostate cancer cases and reported higher fasting insulin and insulin resistance in patients especially older than 65 years [9]. In contrast, one study reported no significant difference in insulin secretion; however, they reported reduced insulin sensitivity in prostate cancer group [10]. Kim et al. reported inverse association of insulin-like growth factor-1 with Gleason score, suggesting that prostate cancer develops independently of insulin-like growth factor-1 [11]. Elevated levels of insulin decreases the production of insulin-like growth factor-1 binding proteins (IGFBP-1), which further causes increase in the insulin-like growth factor-1 levels that increase the production of advanced glycation end products, thus promoting carcinogenesis [12]. Insulin also binds to insulin-like growth factor-1 receptor due to highly similar structure and activates the downstream signaling pathway in prostate cells, thereby proliferating cancer cells [13].

Dyslipidemia is directly associated with mortality in elderly prostate cancer patients. Our study also found non-significant elevated levels of total cholesterol, triglyceride, low-density lipoprotein and very low-density lipoprotein and significantly decreased levels of high-density lipoprotein in both benign prostatic hyperplasia and prostate cancer cases compared to controls. Similar findings were reported in some previous studies [14]. Our study also reported association of deranged lipid profile with higher Gleason score and cancer grade. Harraz et al. also reported significant association of serum lipid profile with risk of prostate cancer [15]. Allott et al. reported serum lipids to be a modifiable factor that influence the risk of prostate cancer recurrence [16]. However, they found no evidence to support their findings. Possible association between deranged lipid profile and prostate cancer recurrence might be due to multiple biological pathways through which serum lipids might affect prostate cancer progression [16,17]. Another systemic analysis studying the influence on lipid profile reported significantly higher levels of total cholesterol and triglyceride after one year of androgen deprivation therapy suggesting vital role of cholesterol in promoting prostate cancer [18].

Testosterone is a key growth factor for prostate; however, its concentration decreases with age. We also observed reduced testosterone levels in benign prostatic hyperplasia and prostate cancer group compared to control and a non-significant negative association of testosterone with prostate cancer Gleason score and grade. Several studies associated lower testosterone levels with increased severity of prostate cancer. However, large number of studies did not find clear association of prostate cancer severity with lower testosterone levels [19]. In contrast to our findings, one study cohort reported elevated serum testosterone levels with increased risk of prostate cancer [20].

We also compared the levels of serum prostate-specific antigen, insulin, insulin-like growth factor-1 and testosterone with family history, physical activity, diet, smoking and alcohol consumption status in prostate cancer cases and found no significant difference in variables except for physical activity. Prostate cancer patients has adverse effects on quality of life which might be preventable with life style modifications. Physical activity improves insulin sensitivity and might reduce the risk of aggressive prostate cancer progression. Study published by Lin et al. also reported benefits of physical activity in lowering insulin levels [21,22]. Several observational studies suggested an inverse association of smoking with prostate cancer; however, risk of death from prostate cancer increases in smokers. Therefore, inverse association in some studies might be due to low prostate cancer screening which leads to detection bias in smokers [23].

Our study may have certain limitations. Firstly, bias from daily dietary habits, smoking status, alcohol consumption and physical activity may exist and could possibly affect risk of prostate cancer. Secondly, the available information on co-morbidities and drugs were also limited, which could be an important confounder as some prostate cancer patients might already take antiandrogen drugs for benign prostatic hyperplasia treatment before diagnosis of prostate cancer which might had affected the levels of biochemical parameters. Our study had small sample size, thus may not be generalized to other ethnicities; hence, multicenter studies examining the association of lipid profile and serum insulin, insulin-like growth factor-1 levels with benign prostatic hyperplasia or prostate cancer are warranted.

## Conclusions

In conclusion, the result of this study has shown a significant positive association of serum insulin and insulin-like growth factor-1 levels with prostate cancer Gleason score and grade. Insulin levels were also higher in prostate cancer patients with no physical activity which suggests that lifestyle modifications like dietary habits and physical exercise might improve insulin sensitivity that could further reduce the progression to advanced-stage prostate cancer. This study also provides valuable insights into the complex mechanism that contributes to the development and progression of prostate cancer as dyslipidemia and hyperinsulinemia have been associated with prostate cancer progression and could serve as potential biomarkers in assessing the risk of prostate cancer. Further research involving metabolic pathways may lead to more effective treatment for prostate cancer ultimately improving cancer prognosis.
